# Patients’ Perspectives on the Use of a Newly Developed “Patients’ Guide for Doctor’s Visit”: DocVISITguide

**DOI:** 10.3390/ijerph20146414

**Published:** 2023-07-20

**Authors:** Rebecca Jaks, Elena Guggiari, Saskia Maria De Gani, Dunja Nicca

**Affiliations:** 1 Careum Center for Health Literacy, Careum Foundation, 8032 Zurich, Switzerland; elena.guggiari@careum.ch (E.G.); saskia.degani@careum.ch (S.M.D.G.); 2 Careum School of Health, Kalaidos University of Applied Sciences, 8006 Zurich, Switzerland; 3 Epidemiology, Biostatistics and Prevention Institute, University of Zurich, 8001 Zurich, Switzerland; dunja.nicca@uzh.ch

**Keywords:** health information, healthcare, patients, doctor-patient communication, evaluation, health literacy

## Abstract

During doctor’s visits, fundamental decisions regarding a patient’s diagnosis and therapy are taken. However, consultations often take place within a limited time frame and are characterized by an asymmetric interaction. Therefore, patients’ questions can remain unanswered and concerns unspoken. A "Patients’ Guide for Doctor’s Visit" (DocVISITguide) was developed to prepare patients for their visits, supporting them to take an active role in the communication and leave the consultation well-informed. This paper describes the development of the DocVISITguide and its first small-scale evaluation based on a sequential explanatory mixed methods design from the patients’ perspective. For this purpose, a small sample of patients completed a pre- and post-questionnaire, and two in-depth interviews were conducted. Most participants found the DocVISITguide easy to understand. The guide helped them to take a closer look at their own health situation and be better prepared for the visit. More than three-quarters (82%) of the participants would probably use the guide again, and all (100%) would recommend it to family and friends. However, some patients felt unsure about using the guide within the consultation and showing it to their physician. To counteract this uncertainty, physicians should be actively involved in the use of such guides in the future.

## 1. Introduction

According to the most recent data, 71% of the people living in Switzerland consulted a general practitioner at least once during the past year, while the number of consultations increased with patients’ age [1]. Furthermore, 43% of them consulted a specialist at least once [1]. These data indicate that a majority of Swiss residents are in regular exchange with a general practitioner, which, in turn, is a key opportunity for both patients and health professionals to interact in regard to important decisions related to patients’ health and well-being [2,3]. Concretely, for the patients, doctor visits represent a central source of information to self-manage their illness as well as to adopt preventive and health-promoting behaviors [3]. For general practitioners and specialists, on the other hand, such consultations constitute an important occasion for obtaining useful information on patients’ expectations, symptoms and concerns, which play a crucial role in diagnosis, treatment and prevention purposes [2]. Furthermore, general practitioners can support their patients with regard to orientation and coordination in the health system, referring them to further service resources [3]. This is of importance since recent national data on health literacy shows that there is a huge need from the patients’ side for support in navigating the health system [4]. Furthermore, the same study revealed that almost half of the population living in Switzerland (49%) reported low health literacy and hence difficulties in dealing with health information, whereas specific population groups tend to have a lower health literacy than others [4]. In Switzerland, as well as in other European countries [5,6], such vulnerable groups include people with a poor health status and with one or more long-term illnesses, low socio-economic status and lower education. Therefore, these vulnerable groups might experience even greater challenges when making decisions for their own health or when consulting health professionals. Health literacy, i.e., the knowledge, motivation and competencies to access, understand, appraise and apply health-related information, is a crucial resource for people to be able to make decisions in regard to their own health in everyday life [7]. Such decisions often take place in interaction with or after visiting health professionals [8]. Therefore, sufficient health literacy is key for patients to better benefit from doctor visits and achieve better health outcomes [9]. Likewise, it is important that health professionals are able to strengthen the health literacy of their counterparts [10,11]. 

The communication between patients and doctors can be a challenging interaction for several reasons, particularly in the case of multiple chronic conditions [12]. On the one hand, physicians need to collect enough information for clinical reasoning and to determine a patient’s health issue [13], often under circumstances of limited time for these ambulatory doctor visits [14]. The lack of time poses the following important challenges: for example, under time pressure, there seems to be a risk that physicians ask fewer questions concerning patients’ symptoms and concerns, conduct a less thorough clinical examination and can offer less advice for a healthy lifestyle [15]. On the other hand, the lack of time during doctor visits can also prevent patients from asking questions, with the consequence that concerns regarding their diagnosis, treatment and other issues related to their health remain unspoken [16]. If the communication between patients and physicians is too asymmetrical, expressed by the absence of patients’ involvement in the decision-making processes [17,18] and by the use of unclarified medical jargon [19], negative consequences for patients’ health can occur [16]. In addition, decisions about the treatment strategy might be made on the basis of insufficient information, and the reasons for the importance of adhering to this strategy may not be understood by the patient [20]. This can consequently result in less adherence to medical therapies [21], and patients might remain unsatisfied with the consultation [18]. 

So far, most measures to improve the interaction between patients and doctors in the context of ambulatory doctor visits have mainly focused on a shared decision-making approach and on sensitizing physicians to the potential asymmetry of the conversation and its consequences [22]. However, patients need to be empowered to use the time during the consultation in a goal-oriented way and to take an active role in the communication (e.g., by asking questions and obtaining comprehensible information) in order to leave the consultation well-informed [23]. Moreover, improving the communication within ambulatory doctor visits can have several benefits for patients, e.g., improving patients’ outcomes and their perceptions of their control over their own health, their recall of information and their adherence to medical recommendations [24].

For example, to support the communication of patients with their doctors, question prompt sheets have been developed to enhance patients’ capabilities to take an active role in medical consultations by encouraging them to ask questions about treatment options. Such question prompt sheets can help to increase the number of questions asked by patients during consultations [24,25,26,27,28] and to improve patients’ satisfaction with the medical consultation [26,27]. The support tools also show the potential to encourage physicians to provide more health-related information to their patients [28]. However, the available tools and resources for supporting patients during consultations were mainly developed in countries other than Switzerland. To our knowledge, no widespread tools have been available in Switzerland so far, and especially have not been evaluated with patients interacting with the Swiss health system. Furthermore, many of the existing resources focus on the consultation itself and do not explicitly include the preparation phase and the follow-up after the consultation.

Hence, with this project, we wanted to develop a culturally sensitive and evidence-informed guide for doctor’s visits for the population of the Canton of Zurich, Switzerland, including the following three phases: pre, during and after consultation. The overall aim of this paper is to describe the development and first small-scale evaluation of the patients’ perspective on the “Patients’ Guide for Doctor’s Visit” (DocVISITguide) by using a sequential explanatory mixed methods design. Concretely, this involved collecting quantitative data on the acceptability, feasibility and adoption of the developed guide and then explaining the quantitative results with in-depth descriptions of patients’ experiences with the guide. 

## 2. Materials and Methods

**Figure 1:**
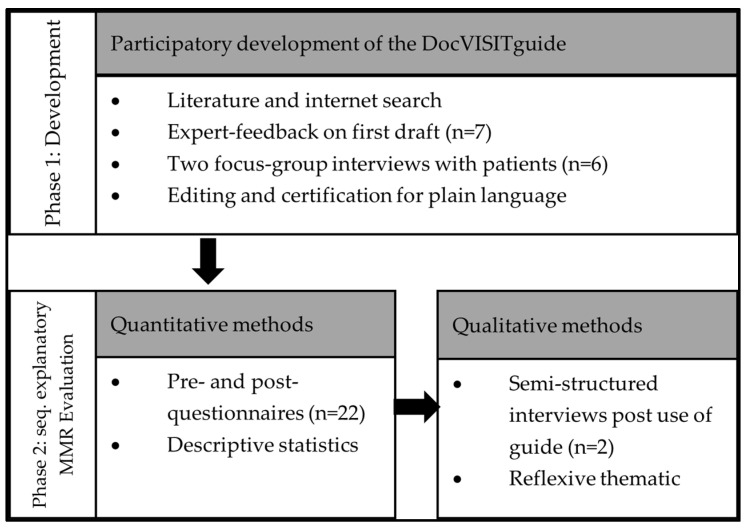
Development of a “Patients’ Guide for Doctor’s Visit” and its evaluation based on a sequential explanatory mixed methods design.

This project includes a participatory approach to develop a Patients’ Guide for Doctor’s Visit (DocVISITguide) (phase 1) and its first small-scale evaluation (phase 2) following a sequential explanatory mixed methods design [29]. The development of the DocVISITguide included (a) a literature and internet search, (b) feedback from patients and professional experts and (c) a certification for plain language, while the evaluation phase included (a) a pre- and post-questionnaire followed by (b) semi-structured interviews (Figure 1). The methods and results are reported along these two project phases. 

### 2.1. Phase 1: Development of the Patients’ Guide for Doctor’s Visit (DocVISITguide)

The development of the DocVISITguide started in 2019 as a practice developmental project within the “Health Literacy Zurich” program. The program is hosted by the Department of Health of the Canton of Zurich (Gesundheitsdirektion Kanton Zürich) and Careum with the aim to improve health literacy within the population of the Canton of Zurich, Switzerland, through a series of research and practice developmental projects.

#### 2.1.1. Literature and Internet Search

For the development of the DocVISITguide, we reviewed the literature on patients’ guidance for visiting healthcare professionals and performed an internet search for already existing tools in German and English.

The results of the literature review were used to formulate the following core principles and quality criteria for the development of such a guide applicable to the entire resident population of the Canton of Zurich over the age of 16: (a) support patients before, during and after the doctor’s visit, (b) be as generally applicable as possible in terms of content, i.e., be usable for all patient groups across all diseases, (c) be adaptable to individual needs of patients and the requirements of a physician consultation, (d) focus on a few core messages and (e) enable a low-threshold use through language and design. 

The internet search provided materials such as checklists and guidelines available for preparing doctor’s visits. A total of 17 tools were identified. They ranged from one-page downloadable documents to elaborate websites with the possibility to compile individualized forms and download supplementary material such as videos or comprehensive brochures. Each of the tools identified was rated based on strengths and weaknesses. Among those we found, nine of them were rated as too comprehensive, with a different focus, disease-specific, disease-oriented or for a different target audience. The remaining eight tools were rated as good or very good but still did not entirely cover all pre-defined core principles. Therefore, we used some information from these tools as a starting point, adapted it for our purpose and developed the first draft of the DocVISITguide. 

#### 2.1.2. Expert-Feedback and Focus Groups with Patients 

The draft version of the DocVISITguide was adapted based on feedback from experts, including professionals and patients.

Seven professionals with expertise in one or more of the following fields were recruited based on a stakeholder analysis: ambulatory medicine, public health, migrant health, patient-safety and/or patient-provider communication. They received the draft version of the DocVISITguide and were asked to provide written comments on how they evaluate the content, the language and comprehensibility as well as to provide open feedback. The written feedback was summarized. This was followed up by a short telephone interview with experts, aiming to clarify specific issues and reach consensus on overall feedback. Feedback was then integrated into the draft version of the DocVISITguide. 

The patient perspective on the draft version was included by means of two focus group interviews with three participants each. The aim was to stimulate discussions among patients or potential patients. The first group was purposefully recruited in a general practitioners practice, focusing on diversity in relation to disease status, gender, age and education. The second group was purposefully recruited outside the health setting focusing on persons of different ages and genders as well as such without current health problems or with chronically ill relatives they care for. We assumed that the two diverse groups would allow a heterogenous perspective on the guide. The focus group discussions were led by a leader of the program. In the beginning, both groups received the DocVISITguide and time to read it before the focus group interviews started. During the interviews they were asked to discuss the general comprehensibility, in particular, unclear contents and formulations, comprehensibility of the use of the tool, shortening options and additions and wishes regarding design and content. Patients’ feedback from both group interviews was mapped during the meeting and summarized, and integrated into the DocVISITguide. 

#### 2.1.3. Editing and Certification for Plain Language

The final version of the DocVISITguide was edited for plain language in order to make the guide understandable for as many people as possible. The language revision was made through an organization in Switzerland (Pro Infirmis), where professional copywriters, as well as review teams, simplified the texts according to the rules of plain language. The DocVISITguide received the official label of “leichte Sprache” (plain language), meaning that the guide follows the rules for easy language and that examiners from the target audience understood the text. 

### 2.2. Phase 2: Sequential Explanatory Mixed Methods Design 

For the evaluation of the DocVISITguide, a sequential explanatory mixed methods design has been used. This involved collecting quantitative data with a pre- and post-questionnaire first and then explaining aspects of the quantitative results with qualitative data collected via interviews.

No ethical approval was needed for the evaluation of the DocVISITguide as it was considered as a practice developmental project. Nevertheless, prior to participation, all participants received written information on the evaluation process and the opportunity to ask questions. Information was provided on aim of the project, procedures, voluntariness of participation and the right to withdraw any time, as well as on data protection measures. 

#### 2.2.1. Sample and Recruitment

For the quantitative questionnaire, we planned to recruit a convenience sample of 30 participants from organizations with a diverse patient population. Within the frame of this practice developmental project, we were interested in implementing new practices timely in a step-wise process and with limited resources. This sampling approach allowed the first small-scale, descriptive feedback on patients’ perspectives with the use of the guide. For generalizable results, a sample almost equal the Swiss population would be needed, given that the DocVISITguide applies to all patients visiting medical ambulatory care, which applies to more than 70% of the Swiss population each year [1]. Inclusion criteria were the following: to be able to speak German, to be 18 years and older, to live in Switzerland, to have a doctor’s visit planned in the near future (approximately 4–6 weeks), to be willing to complete a survey before and one after the doctor’s visit and as a matter of course be willing to use the guide beforehand and ideally use it also during the consultation. The reason for their appointment was not relevant to participation.

Participants were recruited through a large Swiss patient organization via their helpline. The organization’s helpline serves patients with diversity in socio-economic status and place of living (urban, rural areas). However, due to the corona-pandemic, the number of calls as well as medical visits had decreased. We therefore involved two additional patient organizations in the recruitment process, which resulted in nine additional participants. To further improve the recruitment, we approached participants directly in the waiting room of a large general practitioners’ practice. The practice is located in an urban setting serving patients with diversity in socio-economic status. Nevertheless, even this approach has led to only two more patients. For this reason, we decided to spread a call for participation through our LinkedIn channel as well as our newsletter and those of other relevant partners. All these recruitment strategies resulted in a total sample of 22 patients who completed both questionnaires. 

For the qualitative in-depth interviews, we had planned to recruit a sub-sample of four to six people from the quantitative sample. Accordingly, via e-mail, we contacted the 22 participants who had completed the quantitative pre- and post-questionnaire and asked them to take part in interviews (telephone or in-person). We were able to conduct interviews with two participants. 

#### 2.2.2. Data Collection

To assess the acceptability, feasibility and adoption of the DocVISITguide from the point of view of individuals, a pre- and a post-questionnaire was developed. The questionnaires were oriented toward implementation outcomes, specifically acceptability, feasibility and adoption [30]. The pre-questionnaire mainly covered questions on acceptability of the guide. It included 28 questions divided into the following three main sections: questions about the person (7 questions), questions about the guide such as the first impression, comprehensibility, importance of use (17 questions) and questions about the context of the medical visit (4 questions). To be able to guarantee anonymity and simultaneously to allow to match the two questionnaires and to know if a person completed both surveys as requested, we asked three questions that permitted the generation of a unique personal code that participants could easily recreate. The post-questionnaire covered feasibility and adoption with 14 questions focusing on their use of the guide, their perceptions of specific parts as well as their assessment of its applicability, usefulness and potential future use. 

Participants could either complete the pre- and post-questionnaires by means of computer-assisted web interviews or with paper and pencil. Data collection took place between May 2021 and June 2022. 

For the qualitative part that took place after the quantitative pre- and post-assessment, we developed and used a semi-structured interview guide with open-ended questions. This included especially questions on participants’ experiences with the guide and perceptions when using it in interaction with physicians. Of the two participants that accepted our invitation, one interview was conducted over a video conference system, while the other was conducted in person. Both interviews were held in German or Swiss German, lasted approximately 30 min and data were audio recorded.

#### 2.2.3. Data Analysis

For data analysis of the quantitative data, we used IBM SPSS Statistics 27 (IBM Corp., Armonk, NY, USA). Descriptive analyses were performed to characterize the study population and summarize patients’ evaluation of the guide regarding acceptability, feasibility and adoption. For the analysis of the qualitative data, we used six steps oriented toward reflexive thematic analysis, whereas interpretation was limited due to the small data set [31]. In the first step, we familiarized ourselves with the data by listening to the audio data and taking first notes. In the second step, we coded the data set into meaningful segments, including analytical descriptions for each. In the third step, we identified themes by compiling clusters of codes that seemed to share core ideas. In the fourth step, in order to organize the inductively developed themes into a central organizing concept, we compared the themes with the results of the quantitative data set and used a joint display to visualize similarities and differences in qualitative and quantitative data. In steps 5 and 6, we made sure that each qualitative theme was clearly demarked and built around a core concept and that quotations were accurate and illustrative. Given that we had only two participants, we made sure that similarities and differences in experience between the two “cases” became visible. With this process, we focused on inductive thematic saturation, related to the emergence of new codes and themes in the limited data set of two “cases”.

## 3. Results

### 3.1. Description of the DocVISITguide

The DocVISITguide has a length of six pages and is divided into the following four sections: (1) front cover, (2) before the visit (preparation), (3) during the visit (accompaniment) and (4) after the visit (follow-up).

The front cover briefly states why such a guide can be beneficial for patients and gives an overview of the content. This is followed by the second section, dedicated to the preparation phase before the doctor’s visit. This includes three useful tips for users on how to best prepare for their doctor’s visit (e.g., that they can bring a trusted person with them), a small section containing some examples of claims that they can tell their doctor (e.g., “these are my complaints”, “this is better or worse since my last visit” or “these are effects and side-effects of my medication”), as well as central questions to prepare for the visit. These questions cover matters such as what health-related issue they have, what they can do about it, what they need to know about possible treatments, what happens next and where they can find information and support. Concrete questions are, for example, “What is the name of my disease (diagnosis)?”, “What are the treatment options for my disease?” or “What consequences does the treatment have on my everyday life?”. The last part of the preparation phase is a space where notes can be made. This is divided into the following two parts: “this is what I want to say to my doctor” and “this is what I want to ask to my doctor”. The third section during the doctor’s visit contains five tips as well as a short explanation of why they are important and can support patients during the consultation. For example, “allow yourself enough time for a decision” as “you have a say in your treatment. You can also talk to someone else about it.”. The DocVISITguide ends with a part dedicated to the time after the doctor’s visit and what happens next. The guide identifies important points for patients following the doctor’s visit, which should encourage them to follow up on the appointment, such as “When is my next visit to the doctor?”, “Have I forgotten an important question or information?”, “Would I like to call the practice again and ask about it?” or “With whom would I like to discuss open questions or decisions?”.

### 3.2. Use of the DocVISITguide before and after the Doctor’s Visit

#### 3.2.1. Sample Characteristics

The quantitative survey was filled in by 22 participants. Their socio-demographic characteristics are summarized in Table 1. They were mostly female (73%) and between 31 and 40 years old (31%). For most participants (88%), German is their first language and half of them have an educational background in the health sector. 

The two participants of the qualitative interviews (sub-sample) were a male and a female, between 31 and 40 years. One of them lives with a chronic disease and has expertise in self-management for several years, whereas the other went to the doctor for an acute orthopedic problem. Both participants have had some negative experiences with their doctors in the past. They explained that they were motivated to take part in the interviews because they were curious to know more about the DocVISITguide and in order to reflect and talk about their experiences with doctor’s visits.

#### 3.2.2. Quantitative Results

In the first step, the participants were asked some questions about the context of their doctor’s visit. During the last 12 months, participants had visited a doctor with a median of 5.5 times (range = 30). Most participants (45%, 10 out of 22) visited a specialist (other than a gynecologist) or a GP/family doctor (41%, 9 out of 22). The main reason for the visit for most of the respondents (59%, 13 out of 22) was a chronic condition. Regarding the application of the guide, 77% (17 out of 22) of the participants did not inform their health professional about it. 

**Figure 2:**
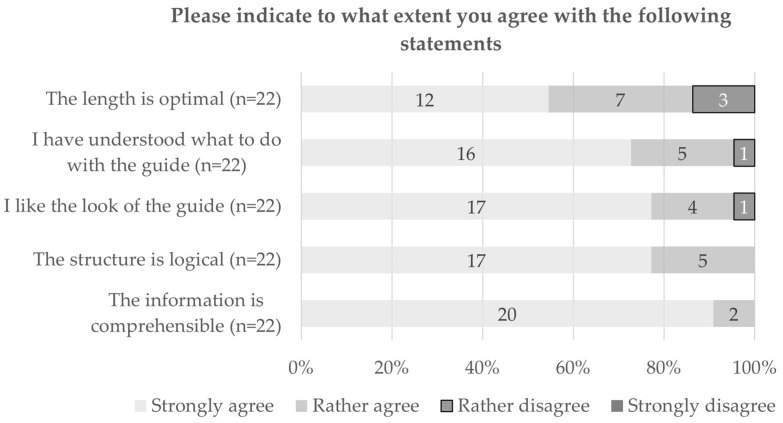
Level of agreement with statements on comprehensibility and content of the guide.

When it comes to the questions on the guide itself, most of the respondents (95%, 21 out of 22) liked the guide at first glance. The content coincided (very) strongly with the expectations of 77% (17 out of 22) of the respondents, while 5% (n = 1) found that it did not match their expectations at all. Further questions on the comprehensibility and content of the guide showed that the structure was considered logical and the information understandable to all participants (100%, 22 out of 22) (see Figure 2). There was less agreement regarding the length of the guide, where 15% (22 out of 22) of the participants stated that it was not optimal. Based on the answers in the open comment section, it seemed that the DocVISITguide was considered a bit too long.

**Figure 3:**
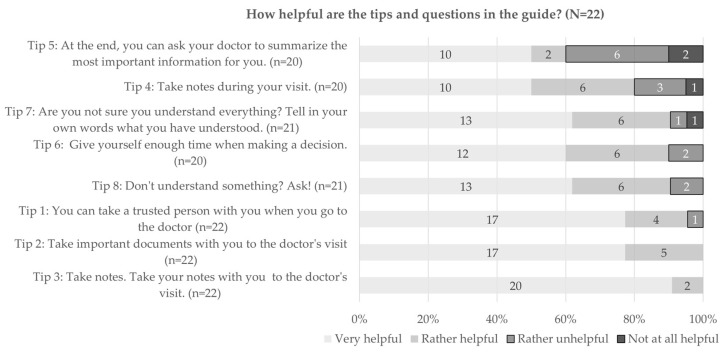
Usefulness of tips in the guide for preparation.

A further question concerned the usefulness of the tips and questions in the guide. Participants rated them, in general, as (rather) helpful, with values varying between 60% (12 out of 20) and 100% (see Figure 3). Tip 5 “At the end you can ask your doctor to summarize the most important information for you”, was considered less useful compared to others. All participants also agreed that making notes beforehand is very useful, and 91% were of the same opinion regarding the questions contained in the DocVISITguide.

Participants were also asked to report which of the tips they planned to apply before or during the visit. Between 81 (17 out of 21) and 95% (21 out of 22) of participants stated to be (rather) sure to use tip 2 “Take important documents with you to the doctor’s visit”, tip 3 “Take notes. Take your notes with you to the doctor’s visit”, tip 6 “Give yourself enough time when taking a decision”, tip 7 “You are not sure you understand everything? Tell the doctor in your own words what you have understood” and tip 8 “You don’t understand something? Ask!”.

**Figure 4:**
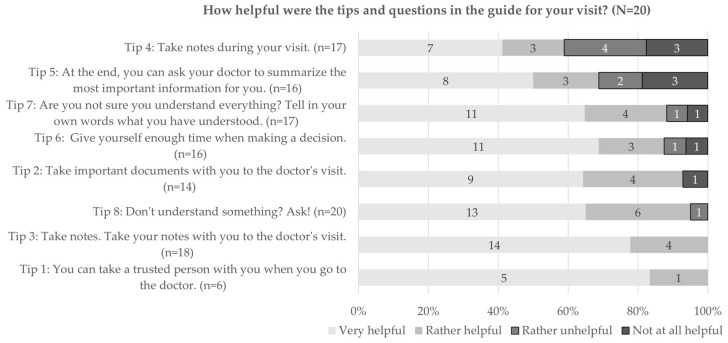
Usefulness of tips in the guide for their visit.

The results show certain discrepancies between planned and actual behavior. In total, 91% (20 out of 22) of the participants stated to have used at least some parts of the guide during their visit. These participants were also asked how useful each tip was for their visit, and most of them felt that the tips were helpful (see Figure 4). Less agreement was found for tip 4 “Take notes during your visit” and tip 5 “At the end, you can ask your doctor to summarize the most important information for you”, where 41% (7 out of 17, tip 4) and 31% (5 out of 16, tip 5), respectively, declared that these tips were rather unhelpful or not at all helpful. Comparing the distribution regarding the usefulness of the tips before and after the visit, we see a similar picture—those tips that were considered more useful already before the visit were also considered more useful afterward.

After the doctor’s visit, 75% (15 out of 20) of the participants stated that they had used the notes made beforehand during their appointment. And both before and after the appointment, all participants (100%) agreed that taking notes was helpful.

The results further show that the majority (77%, 17 out of 22) of the respondents were likely to use the guide again in the future. This number slightly increased after the doctor’s visit (82%, 18 out of 22). All the participants, both before and after the doctor’s visit, affirmed that they would recommend the guide to family, friends and acquaintances. 

**Figure 5:**
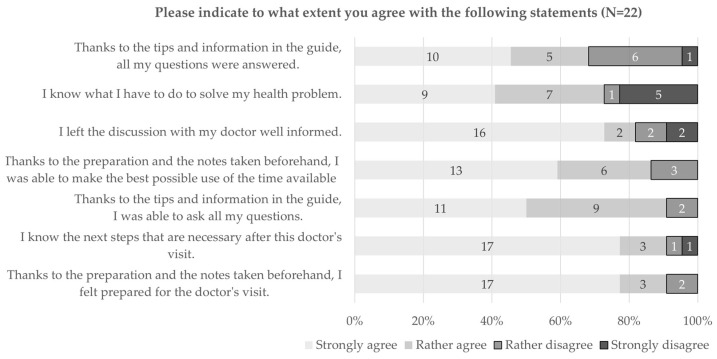
Level of agreement with different statements about the guide and the preparation phase.

Lastly, the results indicate that most of the participants felt prepared for the visit (91%, 20 out of 22) and were able to use the available consultation time at its best (86%, 19 out of 22), thanks to the preparation and the notes taken beforehand (see Figure 5). Furthermore, due to the tips and information in the guide, 91% (20 out of 22) were able to ask their questions; however, 32% (7 out of 22) did not get all their questions answered. Most of the participants left the discussion well-informed (82%, 18 out of 22), and 73% (16 out of 22) knew what to do to solve their health problems.

#### 3.2.3. Qualitative Findings

The quantitative results across three dimensions (acceptability, feasibility and adoption) informed the structure of the inductively generated qualitative themes based on two case studies. The experiences of two patients with the guide are presented with a joint display in Table 2 and are divided into four themes, illustrated with corresponding quotes. 

## 4. Discussion

This practice developmental project resulted in the DocVISITguide aiming to support patients before, during and after their doctor’s visit, and specifically by using the available consultation time in the best manner possible and by taking an active role in the communication via asking questions and following up on the issues discussed during their visit.

The results of the evaluation indicate that participants, who were predominantly well-educated and familiar with the health system, overall liked the content of the DocVISITguide, rated its feasibility as good and would use it again in the future. Participants would also recommend it to their family, friends and acquaintances. Concerning the acceptability of the DocVISITguide, there is a potential discrepancy between the quantitative findings with high acceptability values and the qualitative results. The two in-depth interviews showed that at the beginning, participants were rather skeptical about the DocVISITguide and expectations about it were rather low. They believed that it would not bring them anything new compared to what they already knew or had done so far. However, they still decided to participate in the evaluation and to use the DocVISITguide—probably driven by curiosity about it or because of their past experiences in their interactions with health professionals. Furthermore, they were sensible about the topic of doctor-patient communication and recognized the relevance of the project. Such results indicate that overcoming patients’ skepticism and convincing them to use the DocVISITguide can be quite challenging, despite their familiarity with the health system. Furthermore, the recruitment barriers we experienced might not only relate to the situation around COVID-19 but also relate to such skepticism. These barriers are challenging but could be overcome to some extent if patients are introduced to the DocVISITguide and its benefits directly by health professionals, health organizations or patient organizations they trust and not by an unknown third party or anonymous institution.

The quantitative results also showed that participants gave quite high values to the feasibility in respect to the preparation for the doctor’s visit and to the use of the guide. In particular, most of the participants reported that the guide was understandable and helpful in their preparation for the visit and the procedure during the consultation. These results suggest that despite its generic approach, the DocVISITguide can be a useful tool for diverse patients to prepare for their doctor visits. Further research would be needed to understand potential differences across specific patient groups, in particular those with low health literacy [4]. 

The adoption of the DocVISITguide had positive effects on the participants by giving them useful tips and information or by reassuring them that what they were already doing was in the right direction for a successful doctor’s visit. Applying the DocVISITguide enabled participants to think more deeply about their own health situation and to reflect thoroughly on what they wanted to discuss with and ask their doctor about. These findings confirmed the positive effects already found in previous interventions aimed at improving patients’ communication with doctors, such as an increase in question-asking, taking an active patient role, a better recall of information and patient satisfaction [24,26,28]. However, previous evidence has been mixed, and findings have not been always consistent [28]. More research on the outcomes of interventions aimed at improving patients’ communication with doctors is therefore needed. The results of the present evaluation also showed that, before the visit, participants stated to plan to use certain tips and information from the DocVISITguide for their next doctor’s visit. These initial intentions and the actual behavior, however, partly diverged. These results may have come into play as the DocVISITguide, with its aim of trying to reach as many people as possible, is probably not suitable for all situations, or the patients need further encouragement to actually apply them. Nonetheless, the use of the tips and information contained in the DocVISITguide might depend on the reasons behind the doctor’s visit and on the specific patient’s situation. A person who is just attending a routine control probably has different questions and needs and especially less experience in communicating them than someone with a complex chronic illness and who is frequently in contact with health professionals and confronted with the health system in general [32]. Another reason for the discrepancy between initial intentions and actual behavior could also be related to the limited time available for the consultation [14] and hence for asking questions, even though in Switzerland, this issue seems to be not as worse compared to other countries [33].

Importantly, the results from the two qualitative interviews cannot be generalized, but provide the following important, unexpected insight: participants did not want to show and actively use the DocVISITguide in front of their physician and preferred to not take it with them into the consultation room. During the qualitative interviews, insecurity and shame were mentioned as reasons for not applying or taking the DocVISITguide with them. This insecurity to use the DocVISITguide openly during consultations might be a result of years of asymmetric relationships between patients and physicians [17,22] that have led to general mistrust in this respect. Not having the information with them during the visit may have also led to the patients forgetting some of the points noted beforehand in the DocVISITguide and, consequently, leading to a mismatch between intention (“I plan to use the tips”) and actual behavior (“I used the tips”). Since this aspect is important for the further scale-up potential effectiveness of the DocVISITguide, it should be further explored.

Given that building trust on the individual patient–professional level is an issue, it would be beneficial if health professionals, and especially physicians, were involved in the use of tools like the DocVISITguide and would promote it among their patients. This could reassure patients to actively use the DocVISITguide during their future doctor’s visits, allowing them to ask their questions and concerns and take an active role. A recent qualitative study analyzing patients’ experiences with question prompt lists [34] also found that patients would feel reassured in using such tools if doctors normalized, endorsed and recommended them. Since health professionals are in regular contact with patients and are an important point of contact for health issues and general questions [3], the support of the use of a DocVISITguide would also support patients in dealing with health information and, consequently, also strengthen the health literacy of their patients. In particular, the use of a DocVISITguide could improve the interaction between physicians and vulnerable groups of patients with insufficient health literacy [4,6].

We acknowledge that the development and first small-scale evaluation of the DocVISITguide is not without limitations. Ideally, more diverse participants should have been involved in the evaluation of the guide. The project was conducted during the COVID-19 pandemic, when doctor’s visits were reduced to the minimum [35,36], and patients were probably less willing to participate. Additionally, the discussed skepticism in respect to patient–provider communication with the guide has led to a difficult recruiting process and a smaller, less diverse sample than planned. The qualitative interviews, to some extent, expanded the quantitative findings, explaining in more detail what the perceived barriers to using the guide during doctor’s visits could have been. However, a larger sample would have allowed a richer description of themes. 

Nevertheless, the participatory development of the DocVISITguide led to a guide that is already strongly oriented towards the needs of patients, and the small-scale evaluation, with its rather positive findings, points in the same direction. This highlights that the DocVISITguide with the first adaptations based on the findings is ready to be used for the next phase of evaluation and development. Learning from agile sciences, such small-scale evaluations are important to—(a) guide a user-oriented development of tools early on, (b) bring results back into real-world settings quickly and (c) create a participative learning process before going into more rigorous testing and evaluation [37]. Therefore, the DocVISITguide adapted based on the first patient experiences presented here should go into testing of effectiveness with respect to improving health literacy with a sample that enables the evaluation of sub-groups and different settings. Additionally, implementation outcome evaluation should be integrated and focus on trust and interaction between patients and health professionals.

## 5. Conclusions

Given that the newly developed DocVISITguide, according to the results of its first small-scale evaluation, is well-received by patients to prepare for their doctor’s visit, its advancement and implementation should be carried on. In order to improve its use for patients during their visits with health professionals, implementation strategies should focus on the involvement of professionals in the process. Further systematic evaluation should support these processes.

Results show that the DocVISITguide provides support, in particular, before and during the doctor’s visits, and encourages patients with the therein contained tips and information to take a closer look at their own health situation and to take an active role during the consultation. The DocVISITguide seemed to facilitate a fruitful and well-informed doctor’s visit. Nevertheless, patients might feel insecure about using the guide during the visit and showing or mentioning its use to the doctor. To counteract this feeling and inhibition, the further development of the guide and its implementation should focus more on overcoming patients’ reluctance. One approach could be, for example, to actively involve health professionals in the process of distributing, applying and using such guides together with their patients.

## Figures and Tables

**Table 1:** Characteristics of study participants.

Variable	Quantitative Survey (Paired Cases) n (%)
Total, N (%)	22 (100%)
Gender, n (%)	
Male	6 (27%)
Female	16 (73%)
Age, n (%)	
Younger than 30 years	–
Between 31 and 40 years	7 (32%)
Between 41 and 50 years	3 (14%)
Between 51 and 60 years	2 (9%)
Between 61 and 70 years	5 (23%)
Older than 71 years	5 (23%)
Education, n (%)	
Compulsory school	2 (9%)
Upper secondary level	5 (23%)
Tertiary level	15 (68%)
Education in the health sector, n (%)	
Yes	11 (50%)
No	11 (50%)
Country of birth, n (%)	
Switzerland	18 (82%)
Other country	4 (18%)
Mother language, n (%) *	
German	21 (88%)
French	0 (0%)
Italian	1 (4%)
Other	2 (8%)
Chronic disease, n (%)	
Yes	17 (77%)
No	5 (23%)

Legend: * multiple choice possible.

**Table 2:** Joint display of quantitative and qualitative findings on perception and experience with the DocVISITguide.

Key Quantitative Results	Exploration by Two Case Studies
Expectations before using the guide	
**Acceptability** 95% liked the guide at first glance; For 77% of the participants, the content of the guide matched (very) much with their expectations.	**“What can they teach me”—trying out the guide with low expectations** When learning about the project and guide: Participants explained that they were rather skeptical with regard to their personal benefit by using guide. At the same time, they were curious about its content and potential learnings. “*At first I wasn’t quite sure what it was supposed to be. (...) I thought what can they tell you that I don’t already know? But then I just got through it.*” Another expectation mentioned by the participant with the chronic illness was that the guide would improve physician’s communication with patients, which had been unsatisfying so far. *“When I heard about the pilot-testing, for me it was also a bit of a reason to join, that I thought maybe it was about the doctors or the training of doctors or something like this.* After reading the guide for the first time: Participants felt that many things were common sense or that they already performed many things listed. “*I read it, yes. I read through it, but then I also thought for many things, yes, exactly, I already do it that way, (…) take notes and so on.*”
After having used the guide for preparation and potentially during a doctor’s visit	
**Feasibility:** in respect to preparation with guide All participants found that the information is understandable; 95% knew what they had to do with the guide; 91% felt (very well) prepared for the doctor’s visit thanks to the targeted preparation and the notes taken beforehand. in respect to use of guide 86% were able to make optimal use of the consultation time thanks to the targeted preparation and the notes taken beforehand; 91% were able to ask all their questions thanks to the use of the tips and hints in the guide; 68% stated that all their questions were answered thanks to the use of the tips and hints in the guide.	**“The guide helps against all odds”—experiencing a positive effect through use of the guide** After using the guide for preparation: Participants explained that reading the guide and doing the preparatory work before the visit has been more very helpful than expected. “*[The guide] is really very comprehensive. The preparation is very helpful and allows you to be less at the mercy of the doctor.*” The participant with the orthopedic problem explained that taking the time to think about his situation and what he wanted to know and discuss with the physician helped a lot. “*I don’t think I have ever thought so much before a visit to the doctor. Especially what I want to ask the doctor (...)and simply what is important for me to know*”. The participant with the chronic condition explained that the information contained in the guide reinforced her that she was doing the right thing. “*Before the visit I thought about things on the basis of the guide and (…) it reinforced me in what I am already doing and how I think it should be.*”
**Adoption** 91% used the tips and hints in the guide during their visit. Planned vs. concrete application of specific suggestions of the guide: Use of notes: 82% vs. 75% Use of tips: 72% vs. 53% * Use of questions: 55% vs. 46% * * mean value of all tips	**“Better not to show it off”—not wanting to use the guide during the visit** When remembering the use of the guide: Participants explained that they preferred not to take the guide or the notes they made beforehand to the visit. “To be honest, when I went to the appointment, I didn’t take it with me. I didn’t feel like taking it with me and saying to the doctor * I’m using this guide *.” The participant with the chronic condition specified that she did not want to use the guide in front of the physician, but rather tries to memorize notes. “*I actually hide it, I have my notes with me rather in advance and look at it again in the waiting room and then I already know quite exactly what I want and what I must not forget.*”
*No matching quantitative data*	**“Building bridges”—involving professionals to take full advantage of the guide** When reflecting about their use of the guide: Participants explained that in order to feel confident to use the guide during the consultation, it should be known and valued by professionals. *“For me, it would be important to work with the physicians so that they can assume or ask that you have it [the guide]. For example, it could be placed in the waiting room, so that it comes from the practice. In this way, the patient would not feel “stupid” to show up with such an instrument.*

## Data Availability

The data, as well as the DocVISITguide presented in this paper, are available on request from the corresponding author.
